# Topography-guided photorefractive keratectomy combined with accelerated corneal collagen cross-linking versus cross-linking alone for progressive keratoconus: a long-term prospective cohort study

**DOI:** 10.3389/fmed.2024.1420264

**Published:** 2024-08-12

**Authors:** Zhihao Dai, Ziyuan Liu, Yu Zhang, Yufei Yuan, Yan Liu, Yuexin Wang, Shuo Yu, Yueguo Chen

**Affiliations:** ^1^Department of Ophthalmology, Peking University Third Hospital, Beijing, China; ^2^Beijing Key Laboratory of Restoration of Damaged Ocular Nerve, Peking University Third Hospital, Beijing, China; ^3^Peking University Institute of Laser Medicine, Beijing, China

**Keywords:** topography guided photorefractive keratectomy, accelerated corneal cross-linking, progressive keratoconus, higher order aberrations, vertical coma

## Abstract

**Purpose:**

To comprehensively compare the long-term outcome of the combined topography guided photorefractive keratectomy (TG-PRK) with accelerated corneal cross-linking (ACXL) and ACXL alone in eyes with progressive keratoconus. The analysis focused on the changes in the detailed corneal aberrometric values.

**Methods:**

This single-center, prospective cohort study included 28 patients (30 eyes) of the TG-PRK plus ACXL group and 14 patients (15 eyes) of the ACXL alone group. The mean duration of the follow-up was 44 ± 10.18 months (ranged from 31 to 65 months). The preoperative data and the postoperative measurement data at the last follow-up visit, including demographic data, uncorrected distance visual acuity (UDVA), corrected distance visual acuity (CDVA), manifest refraction, corneal topography, pachymetry, aberrometry and densitometry were analyzed.

**Results:**

The CDVA significantly improved in the TG-PRK plus ACXL group at the last follow-up visit (*p* = 0.006), while no significant improvement was found in the ACXL alone group (*p* = 0.432). The maximal keratometry of the anterior corneal surface (Kmax) of both groups significantly decreased at the last follow-up visit (*p* < 0.05). Compared with the ACXL alone group, the Kmax of the TG-PRK plus ACXL group showed a greater decline (*p* = 0.008). The total corneal aberrations, the corneal lower-order aberrations (LOAs), the corneal higher order aberrations (HOAs), the vertical coma and the spherical aberration (SA) at the 4.0 mm and 6.0 mm zone of the TG-PRK plus ACXL group significantly decreased at the last follow-up visit (all *p* < 0.05). The declines of the total corneal aberrations, the corneal LOAs, the corneal HOAs and the vertical coma at the 4.0 mm and 6.0 mm zone of the TG-PRK plus ACXL group were significantly higher than those in the ACXL alone group (*p* < 0.001).

**Conclusion:**

Compared with ACXL alone, combined TG-PRK with ACXL procedure had a significantly higher reduction in the corneal HOAs and better CDVA, while providing a similar long-term stability and safety. For progressive keratoconus patients with adequate corneal thickness, the combined procedure might be a recommended treatment option.

## 1 Introduction

Keratoconus is recognized as a progressive, bilateral, and often asymmetric corneal condition influenced by both genetic (family history) and environmental factors, such as eye rubbing and nocturnal ocular compression (1). It is now acknowledged that inflammatory factors play a role in the disease (2–4). Characteristically, keratoconus leads to thinning and steepening of the paracentral cornea, which results in progressive irregular astigmatism and may lead to irreversible visual loss (1). Corneal cross-linking (CXL) can increase the mechanical rigidity of cornea, and it is proved to be an effective method to halt the progression of the keratoconus (5). In the standard protocol (also known as “Dresden protocol”), the corneal epithelium was removed and the central cornea infiltrated with 0.1% riboflavin solution was exposed to the Ultraviolet A light at 3 mW/cm^2^ for 30 min (6). Accelerated corneal cross-linking (ACXL) with much higher irradiation were proposed thereafter, and the protocols included 9 mW/cm^2^ for 10 min, 18 mW/cm^2^ for 5 min, and so on (7). Compared with the standard CXL, these protocols offered the similar efficacy but a shorter operative duration (8). However, in general, CXL is not considered as a refractive procedure and may not contribute to the visual improvement.

The earliest attempt to solve the puzzle was the combination of the sequential treatment of topography-guided photorefractive keratectomy (TG-PRK) and CXL (9). An optimized protocol of simultaneous TG-PRK and ACXL was proposed by Krueger and Kanellopoulos thereafter, which was named “Athens protocol” (10). Since then, different protocols on different platforms had been proposed, demonstrating efficacy in delaying disease progression and improving the visual acuity (11–14). In comparison with CXL alone, the combined procedure demonstrated the significantly improved vision, with the similar results regarding postoperative stability and complications (15–18).

Theoretically, the TG-PRK procedure regularized the central cornea of the progressive keratoconus patients, leading to less corneal higher order aberrations (HOAs) (19). This might be the critical reason for the greater visual improvement of the combined procedure. However, only a few studies have assessed ocular or corneal aberrations. Iselin et al. reported a significant improvement of the total corneal HOAs, mainly coma-like aberrations, at 12 and 24 months after the combined procedure in comparison to CXL alone (15). However, only a few aberrometric parameters, including the total corneal aberrations, the corneal lower-order aberrations (LOAs), the corneal HOAs and the coma-like aberrations, were involved in the study. Other corneal HOAs associating with the postoperative visual quality of the patients were not analyzed. There is a need for a more comprehensive and detailed analysis of the aberrations. Furthermore, considering the longitudinal changes of the corneal morphology, the long-term observation is needed.

In this study, we comprehensively compared the long-term outcome of the combined TG-PRK with ACXL surgery and ACXL alone. We focused on the change in the detailed aberrometric values, and hoped to get a comprehensive understanding of the combined procedure.

## 2 Materials and methods

### 2.1 Patients and study design

This was a single-center, prospective cohort study. The study was conducted at Peking University Third Hospital. Written consents were obtained before surgery. Peking University Third Hospital Medical Science Research Ethics Committee approved this study according to the Declaration of Helsinki (IRB No.00006761).

Between October 2015 and March 2021, a total of 42 patients (45 eyes) with the progressive keratoconus were enrolled in this study. The diagnosis of keratoconus was based on the slit lamp examination, corneal tomography and pachymetry findings. The keratoconus was further characterized as “progressive” if either of the following criteria was met: (1) an increase of 1D or more in the maximal keratometry of the anterior corneal surface (Kmax) within 12 months; (2) the astigmatism changes of more than 1 D within 12 months; (3) a decrease of 30 μm or more in the thinnest corneal thickness (TCT) (20).

The preoperative corneal thickness of the enrolled patients met the criteria for the combined procedure, ensuring a TCT of more than 450 μm or a predicted postoperative thickness of stromal bed of more than 350 μm. Exclusion criteria were as follows: (1) Refusal to participate in this study or not available for long-term follow-up; (2) Other types of corneal ectasia, especially the pellucid marginal degeneration; (3) Previous history of corneal surgery, such as keratoplasty and laser vision correction; (4) History of ocular diseases other than keratoconus or ocular trauma; (5) Presence of systemic disease, especially diabetes, autoimmune and infective disease; (6) Pregnant and lactation or planning a pregnancy within 1 year after ACXL.

At the time of cohort entry, patients were stratified according to the preoperative Kmax: (1) Severe subgroup: Kmax values ≥55D; (2) Mild to moderate subgroup: Kmax values < 55 D. Within either subgroup, patients were allocated to the TG-PRK plus ACXL group and the ACXL alone group. The allocation ratio was controlled at 2:1, considering that the study participants would potentially benefit from the combined procedure.

### 2.2 Clinical examination

All patients underwent a comprehensive ophthalmic examination before surgery. We analyzed the preoperative data and the postoperative measurement data at the last follow-up visit, including demographic data, uncorrected distance visual acuity (UDVA), corrected distance visual acuity (CDVA), manifest refraction, corneal topography, pachymetry, aberrometry and densitometry. UDVA and CDVA were converted to logMAR for analysis. Besides, the severity of the corneal haze was evaluated by slit-lamp biomicroscopy at the last follow-up visit. It was graded according to the system reported by Hanna et al. (21). The primary outcome was the aberrometry results at the last follow-up visit, and the secondary outcomes were the visual acuity, the refractive results, tomography, pachymetry, densitometry and postoperative complications.

Corneal tomography and pachymetry were measured by Pentacam HR system (Oculus GmbH, Wetzlar, Germany). Parameters including anterior radius of curvature (ARC), posterior radius of curvature (PRC), Belin/Ambrósio total deviation value (BAD-D), index of surface variance (ISV), index of vertical asymmetry (IVA), keratoconus index (KI), center keratoconus index (CKI), index of height asymmetry (IHA) and index of height decentration (IHD) were used to evaluate the severity of keratoconus. Flat keratometry (K1), steep keratometry (K2), Kmax, TCT, central corneal thickness (CCT) and apex thickness (AT) were collected for the longitudinal analysis.

In addition, aberrometric and densitometric values were obtained at each follow-up visit using the Pentacam. In the Pentacam HR system, the corneal wavefront aberrations were reconstructed from the Scheimpflug corneal tomographic data. Aberrations for the entire cornea over the calculation diameter of 2, 4 and 6 mm were analyzed with the Zernike polynomials (22). A refractive index of 1.3375 were used for the computation of the aberrations. The data included the root mean square (RMS) of the total corneal aberrations, the RMS of the corneal LOAs, the RMS of the corneal HOAs, horizontal coma, vertical coma, horizontal trefoil, oblique trefoil and spherical aberration (SA). The densitometry values for total corneal thickness within the central 0-2 mm cornea and the intermediate 2–6 mm zone were expressed in grayscale units (23).

### 2.3 Surgical procedure

At the initiation of the procedure, the central 9 mm of the epithelium was mechanically debrided after the cornea was exposed to 20% ethylic alcohol for 20 s using a well. All procedures were performed by the same surgeon (YGC).

#### 2.3.1 Topography guided photorefractive keratectomy surgical procedure

Prior to the surgery, a high-quality scan was obtained using Topolyzer Vario (Alcon, United States). The magnitude and the axis of the preoperative corneal astigmatism was automatically calculated based on the Topolyzer topographic results. Given that the goal of the excimer laser surface ablation was to minimize corneal irregularities rather than correct refractive errors, the sphere correction was set to “0” in the preoperative refractive planning. The size of the ablation optical zone was set at 5.0, 5.5, 6.0 or 6.5 mm, and the maximum ablation depth was limited to 50 μm. The cylinder correction was customized for each patient, not exceeding the stromal ablation depth limit. The magnitude of the cylinder correction ranged from 0 to 2.75D, and the axis was consistent with the axis of the preoperative corneal astigmatism. All TG-PRK procedures were performed on the WaveLight EX500 (Alcon, United States) excimer laser. The static cyclotorsion and the chord mu, which is a proxy for the kappa angle, were automatically compensated for (24). Mitomycin C was not used during the surgery. The main parameters of the TG-PRK procedure were presented in Table 1.

**Table 1:** Procedure-related parameters.

	Mean ± SD/Median (IQR)^*^
Optical zone diameter (mm)	5.50 (0.50)
Transition zone diameter (mm)	1.25 (0.00)
Total ablation zone (mm)	8.50 (0.50)
Maximum ablation depth (μm)	40.91 ± 7.59
Central ablation depth (μm)	29.62 ± 7.50
Predicted postoperative residual stromal bed thickness (μm)	374.93 ± 27.01
Attempted sphere (D)	0.00 (0.00)
Attempted cylinder (D)	−1.22 ± 0.79

SD, standard deviation; IQR, interquartile range. *Normally distributed parameters were expressed as mean ± SD, while non-normally distributed parameters were expressed as the median with IQR.

#### 2.3.2 Accelerated corneal cross-linking procedure

Immediately after the TG-PRK procedure or the epithelial debridement, ACXL was performed using a uniform protocol. Before irradiation, the anterior surface of the deepithelialized cornea was thoroughly soaked in 0.1% riboflavin (Peschke M®, Peschke Meditrade GmbH) for 10 min. Afterward, the central 8 mm of the cornea was exposed to the 365 nm ultraviolet-A light. Both groups had underwent the ACXL at 30 mW/cm^2^ for 4 min. At the same time, we continued to apply 0.1% riboflavin drops to the cornea to maintain the riboflavin saturation.

After the surgery, a bandage contact lens was applied. The lens was removed after 7 days when re-epithelialization was completed. All patients were treated topically with 0.5% levofloxacin (Santen Pharmaceuticals, Osaka, Japan) 4 times a day until complete corneal epithelialization. Fluorometholone 0.1% (Santen Pharmaceutical, Osaka, Japan) was applied 4 times a day with weekly tapering for 4 weeks. To reduce the effect of the intraocular pressure on the cornea morphology and prevent the steroid-induced ocular hypertension in the short-term after surgery, all patients were instructed to apply 2% carteolol hydrochloride (Otsuka Pharmaceutical, Shanghai, China) 2 times a day for 4 weeks.

### 2.4 Astigmatic vector analysis

Vector analysis of the manifest astigmatism was performed with the assistance of the ASSORT Group Analysis Calculator (ASSORT Pty. Ltd.) based on the Alpins method (25). All manifest refraction data was converted from the spectacle plane to the corneal plane using a vertex distance of 12 mm. We calculated the following vectors: surgically induced astigmatism (SIA), which represents the astigmatism achieved by the surgery; target-induced astigmatism (TIA), which represents the astigmatic correction that was attempted; angle of error (AE), which represents the angle between the axis of the SIA and the axis of the TIA; difference vector (DV), which represents the vectorial difference between the TIA and SIA vectors; correction Index (CI), which represents the SIA divided by the TIA.

### 2.5 Sample size calculation

We aimed to detect the differences between the postoperative corneal HOAs of the TG-PRK plus ACXL group and the ACXL alone group. Based on the previous clinical trial, the mean postoperative corneal HOAs of the CXL alone group was 0.73 ± 0.36 μm. A decline in the corneal HOAs of 0.33 μm was expected in the TG-PRK plus CXL group (15). The sample size ratio between the two groups was 2:1. A power of 80% was set and the alpha error was set as 0.05. With these settings, we estimated that we needed a total of at least 45 eyes, 30 eyes in the TG-PRK plus ACXL group and 15 eyes in the ACXL alone group. Sample size calculation was performed with G*power software, V.3.1.9.

### 2.6 Statistical analysis

Data analysis was performed with SPSS 24.0 (IBM SPSS Statistics 24.0, United States). Data were graphed using Microsoft Excel 2019 (Microsoft Corporation, Redmond, WA, United States). Normal distribution was tested with the Shapiro–Wilk test and QQ plots. Normally distributed data was expressed as mean ± standard deviation, while non-normally distributed continuous data was presented as median (interquartile range). Among the long-term follow-up, no patients were lost and no data were missing. In each group, the comparison between preoperative and postoperative parameters were performed with paired t-test. Student’s t-test was applied for comparison between different groups. All the continuous variables involved in the comparison were showed to be normally distributed. Categorical variables (e.g., gender) were expressed as number (percentage), and Chi-square test was used for analysis. A *p* value less than 0.05 was considered significant. To further assess the long-term outcomes, we performed the analysis in severity subgroups. Given the limited statistical power, subgroup analysis was considered as exploratory only. The results of the subgroup analysis were presented in Supplementary Table S1 for the readers’ further reference.

## 3 Results

There were 28 patients (30 eyes) in the TG-PRK plus ACXL group and 14 patients (15 eyes) in the ACXL alone group. Two patients underwent bilateral TG-PRK plus ACXL and 1 patient underwent bilateral ACXL alone. The mean duration of the follow-up was 44 ± 10.18 months (ranged from 31 to 65 months). Demographic and preoperative data of both groups were presented in Table 2. There were no significant differences in the baseline characteristics between the two groups (all *p* > 0.05).

**Table 2:** Patient demographic and preoperative data.

Characteristics	TG-PRK plus ACXL group (*n* = 30)		ACXL alone group (*n* = 15)		*P* value
	Preoperative	Range	Preoperative	Range	
Gender (m/f)	19 (63.3%) / 11 (36.7%)		11 (73.3%)/4 (26.7%)		0.502
Laterality (OD/OS)	10 (33.3%) / 20 (66.7%)		6 (40.0%)/9 (60.0%)		0.660
Age at operation (years)	23 ± 7.73	9–45	23 ± 5.98	14–37	0.816
Postoperative follow-up (months)	46 ± 10.06	31–65	41 ± 9.95	32–60	0.132
Visual acuity					
UCVA (logMAR)	0.75 ± 0.37	0.10–1.70	0.88 ± 0.36	0.22–1.70	0.291
CDVA (logMAR)	0.24 ± 0.17	0.00–0.70	0.20 ± 0.15	0.00–0.52	0.480
Refractive results					
Manifest Sphere (D)	−4.96 ± 2.75	−1.25–-11.25	−6.23 ± 3.39	−2.00–-12.25	0.183
Manifest Cylinder (D)	−3.86 ± 1.74	−1.50–-8.00	−4.10 ± 2.52	0.00–-10.00	0.708
MRSE (D)	−6.89 ± 2.97	−2.00–-14.25	−8.28 ± 3.79	−3.00–-14.50	0.183
Topography					
ARC (mm)	6.68 ± 0.44	5.96–7.64	6.86 ± 0.43	6.06–7.74	0.219
PRC (mm)	5.07 ± 0.44	4.24–5.93	5.16 ± 0.44	4.38–6.24	0.503
K1 (D)	45.74 ± 2.62	41.4–51.5	44.71 ± 2.04	41.4–48.2	0.193
K2 (D)	50.02 ± 3.35	43.9–56.8	49.15 ± 2.76	44.8–55.9	0.387
Kmax (D)	55.74 ± 4.90	47.2–64.5	54.83 ± 4.38	45.8–62.4	0.544
BAD-D	7.73 ± 2.43	3.63–12.75	7.23 ± 2.72	3.29–13.65	0.531
Pachymetry					
CCT (μm)	492.10 ± 22.00	450–556	487.87 ± 20.42	456–526	0.537
AT (μm)	483.97 ± 22.10	443–552	480.47 ± 20.16	454–527	0.609
TCT (μm)	476.17 ± 23.79	436–547	473.60 ± 21.26	441–516	0.726
Severity indices					
ISV	80.03 ± 25.02	37–130	77.80 ± 31.79	34–136	0.798
IVA	0.81 ± 0.32	0.28–1.44	0.79 ± 0.45	0.24–1.69	0.848
KI	1.20 ± 0.07	1.09–1.34	1.19 ± 0.11	1.03–1.44	0.711
CKI	1.07 ± 0.04	0.99–1.16	1.06 ± 0.04	1.01–1.13	0.756
IHA	32.16 ± 27.96	0.8–110.0	35.00 ± 30.59	3.0–122.0	0.757
IHD	0.114 ± 0.047	0.034–0.193	0.103 ± 0.061	0.021–0.222	0.527

TG-PRK, topography-guided photorefractive keratectomy; ACXL, accelerated corneal cross-linking; m, male; f, female; OD, right eye; OS, left eye; UDVA, uncorrected distance visual acuity; CDVA, corrected distance visual acuity; MRSE, refractive spherical equivalent; ARC, anterior radius of curvature; PRC, posterior radius of curvature; K1, flat keratometry; K2, steep keratometry; Kmax, the maximal keratometry of the anterior corneal surface; BAD-D, Belin/Ambrósio total deviation value; CCT, central corneal thickness; AT, apex thickness; TCT, thinnest corneal thickness; ISV, index of surface variance; IVA, index of vertical asymmetry; KI, keratoconus index; CKI, center keratoconus index; IHA, index of height asymmetry; IHD, index of height decentration. Data were presented as mean ± standard deviation unless otherwise indicated. A P value of less than 0.05 was considered statistically significant.

Table 3 showed the visual acuity, refractive outcomes, topography and pachymetry at the last follow-up visit. Compared with preoperative values, the CDVA at the last follow-up visit improved significantly to 0.14 ± 0.16 in the TG-PRK plus ACXL group (*p* = 0.006), while no significant improvement was found in the CDVA of the ACXL alone group (*p* = 0.432). No significant improvements in the UDVA and the manifest refraction were found in either group at the last follow-up visit.

**Table 3:** Visual acuity, refractive outcome, topography and pachymetry at the last follow-up visit in the TG-PRK plus ACXL group and the ACXL alone group.

	TG-PRK plus ACXL group (*n* = 30)		ACXL alone group (*n* = 15)		*p*-value^b^
	Last follow-up	*p*-value^a^	Last follow-up	*p*-value^a^	
Visual acutiy and refractive results					
UDVA (logMAR)	0.75 ± 0.29	0.948	0.74 ± 0.30	0.062	0.955
CDVA (logMAR)	0.14 ± 0.16	0.006	0.18 ± 0.13	0.432	0.404
Manifest Sphere (D)	−4.85 ± 3.11	0.759	−7.12 ± 3.15	0.205	0.035
Manifest Cylinder (D)	−3.79 ± 2.16	0.794	−4.12 ± 1.63	0.780	0.632
MRSE (D)	−6.75 ± 3.47	0.745	−9.17 ± 3.25	0.358	0.038
Topography					
K1 (D)	45.20 ± 2.48	0.001	44.65 ± 1.90	0.750	0.450
K2 (D)	49.38 ± 3.58	0.010	48.89 ± 2.74	0.373	0.644
Kmax (D)	52.68 ± 4.96	<0.001	53.87 ± 4.39	0.035	0.435
Pachymetry					
CCT (μm)	470.50 ± 25.34	<0.001	491.80 ± 26.56	0.199	0.012
AT (μm)	459.43 ± 22.48	<0.001	481.73 ± 26.43	0.640	0.005
TCT (μm)	447.37 ± 21.87	<0.001	474.40 ± 27.06	0.742	0.001

TG-PRK, topography-guided photorefractive keratectomy; ACXL, accelerated corneal cross-linking; UDVA, uncorrected distance visual acuity; CDVA, corrected distance visual acuity; MRSE, refractive spherical equivalent; K1, flat keratometry; K2, steep keratometry; Kmax, the maximal keratometry of the anterior corneal surface; CCT, central corneal thickness; AT, apex thickness; TCT, thinnest corneal thickness. Data were presented as mean ± standard deviation unless otherwise indicated. A P value of less than 0.05 was considered statistically significant. ^a^Differences between preoperative and postoperative values. ^b^Differences between two groups at the last follow-up visit.

Compared with the preoperative value, the Kmax of both groups significantly decreased at the last follow-up visit (*p* < 0.05). K1 and K2 decreased significantly in the TG-PRK plus ACXL group (*p* < 0.05), and remained stable at the ACXL alone group (*p* > 0.05). Corneal thickness parameters, including CCT, AT and TCT, decreased significantly in the TG-PRK plus ACXL group (all *p* < 0.001), while remained stable in the ACXL alone group (*p* > 0.05).

**Figure 1:**
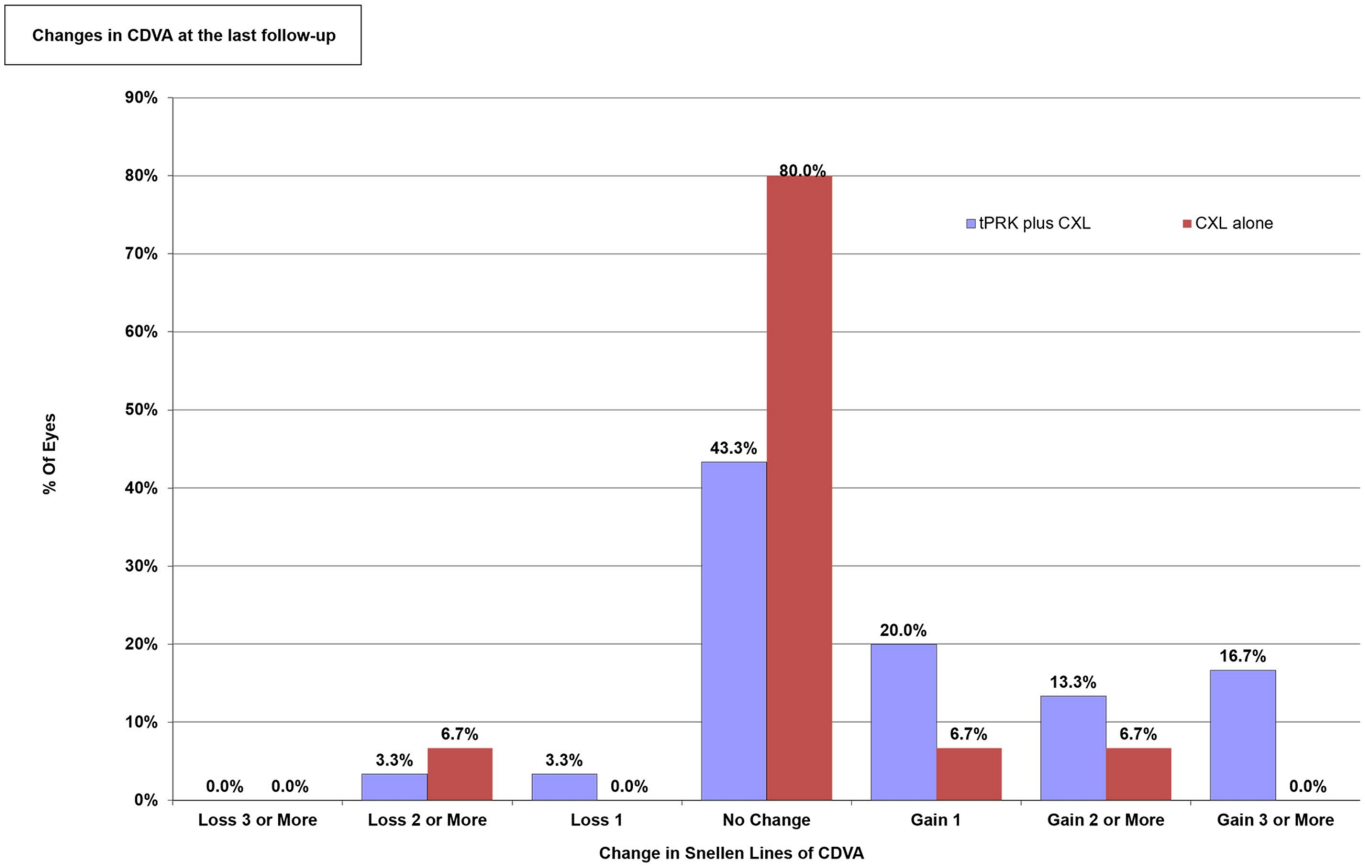
The changes in the corrected distance visual acuity (CDVA) expressed as the percentage of Snellen lines gained or lost. TG-PRK, topography-guided photorefractive keratectomy; ACXL, accelerated corneal cross-linking; CDVA, corrected distance visual acuity.

For both groups, Figure 1 illustrated the Snellen line changes in CDVA from baseline to the postoperative follow-ups. At the time of the last follow-up visit, 9 eyes (30.0%) of the TG-PRK plus ACXL group and 1 eye (6.7%) of the ACXL alone group gained 2 or more lines.

The vector analysis outcomes of the TG-PRK plus ACXL group at the last follow-up visit were shown in Supplementary Figure S1. The mean TIA of the TG-PRK plus ACXL group was 0.74 × 173°, while the mean SIA was 0.18 × 105° (Supplementary Figures S1C,D). The mean DV was 0.87 × 177° (Supplementary Figure S1E). However, there was no significant correlation between TIA and SIA (*p* > 0.05; Supplementary Figure S1A). The AE and the CI had a large variation (Supplementary Figures S1B,F).

**Figure 2:**
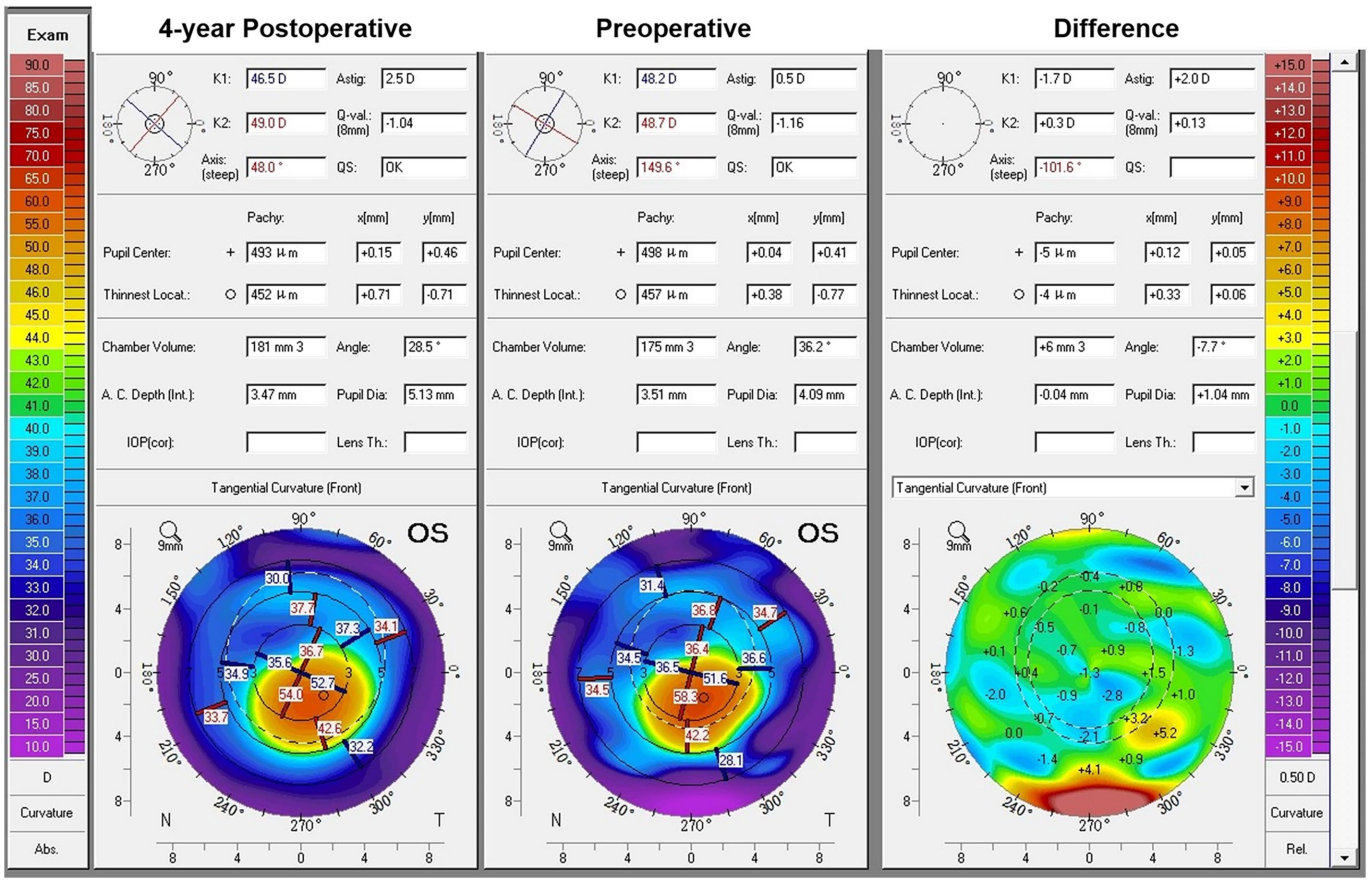
Pentacam Scheimpflug images obtained from the left eye of a 16-year-old male patient who had undergone the combined topography guided photorefractive keratectomy with accelerated corneal cross-linking procedure. Preoperative tangential curvature map showed the keratoconus with the inferiorly decentred cone (middle). Postoperative tangential curvature map at 4-year follow-up visit revealed the flattening and better centration of the cone (left side). The corresponding tangential curvature difference map demonstrated the annular ablation zone located in the paracentral cornea (right side). The laser ablation flattened the cone apex and steepened the superior central cornea by flattening the surrounded section, thus regularizing the morphology of the central cornea.

Representative preoperative and postoperative topography maps of the combined TG-PRK with ACXL procedure obtained by Pentacam were shown in Figure 2. Significant topographic improvement could be seen 4 years postoperatively.

**Figure 3:**
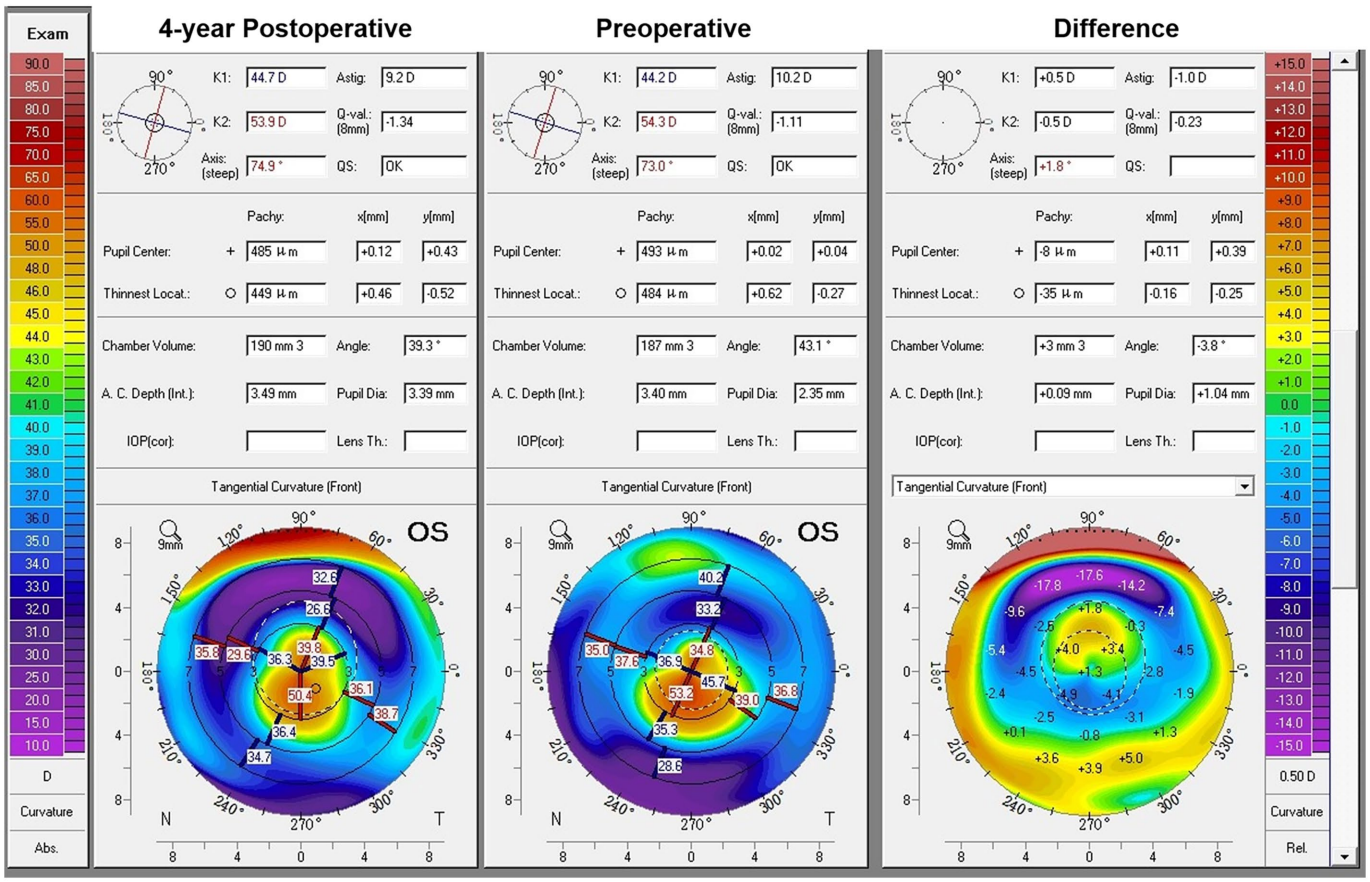
Pentacam Scheimpflug images obtained from the left eye of a 25-year-old male patient who had undergone the accelerated corneal cross-linking alone. Preoperative tangential curvature map showed the keratoconus with the inferiorly decentred cone (middle). No significant alteration in corneal morphology was observed at 4-year postoperative tangential curvature map (left side) and the corresponding tangential curvature difference map (right side).

Representative preoperative and postoperative topography maps of the ACXL alone obtained by Pentacam were shown in Figure 3. No significant changes were observed. Table 4 presented the preoperative and postoperative aberrometric values in various calculation zones for both groups. Compared with the preoperative value, the total corneal aberrations, the corneal LOAs, the corneal HOAs, the vertical coma and the SA at each corneal zone of the TG-PRK plus ACXL group significantly decreased at the last follow-up visit (all *p* < 0.05). The horizontal coma at the corneal zone of 6.0 mm, the oblique trefoil at the corneal zone of 4.0 mm and 6.0 mm also significantly decreased (p < 0.05). In the ACXL alone group, the total corneal aberrations and the corneal LOAs of the 4.0 mm and 6.0 mm zone significantly decreased at the last follow-up visit (p < 0.05). In addition, the corneal HOAs and the vertical coma of the 2.0 mm and 4.0 mm zone significantly decreased (p < 0.05).

**Table 4:** Pre- and postoperative comparison of aberrometric data for the TG-PRK plus ACXL group and the ACXL alone group at the last follow-up visit.

	TG-PRK plus ACXL group (*n* = 30)			ACXL alone group (*n* = 15)		
	Preoperative	Last follow-up	*p*-value	Preoperative	Last follow-up	*p*-value
2.0 mm zone						
RMS total (μm)	0.77 ± 0.33	0.53 ± 0.32	<0.001	0.79 ± 0.36	0.70 ± 0.32	0.130
RMS LOAs (μm)	0.75 ± 0.32	0.52 ± 0.32	<0.001	0.77 ± 0.36	0.69 ± 0.32	0.139
RMS HOAs (μm)	0.16 ± 0.08	0.08 ± 0.06	<0.001	0.15 ± 0.08	0.14 ± 0.07	0.020
Coma 0 (μm)	0.01 ± 0.07	0.00 ± 0.04	0.242	0.00 ± 0.06	−0.01 ± 0.07	0.060
Coma 90 (μm)	−0.14 ± 0.07	−0.04 ± 0.06	<0.001	−0.13 ± 0.08	−0.11 ± 0.08	0.003
Trefoil 0 (μm)	0.01 ± 0.03	0.00 ± 0.02	0.084	0.00 ± 0.03	0.01 ± 0.02	0.126
Trefoil 30 (μm)	0.00 ± 0.02	0.01 ± 0.03	0.086	0.02 ± 0.04	0.02 ± 0.04	0.956
Spherical aberration	−0.01 ± 0.02	0.00 ± 0.01	0.002	−0.01 ± 0.01	−0.01 ± 0.01	0.546
4.0 mm zone						
RMS total (μm)	3.94 ± 1.59	2.40 ± 1.36	<0.001	3.82 ± 1.57	3.46 ± 1.58	0.005
RMS LOAs (μm)	3.82 ± 1.54	2.34 ± 1.33	<0.001	3.70 ± 1.51	3.35 ± 1.51	0.009
RMS HOAs (μm)	0.99 ± 0.44	0.48 ± 0.33	<0.001	0.94 ± 0.50	0.87 ± 0.49	0.029
Coma 0 (μm)	0.08 ± 0.42	0.01 ± 0.26	0.094	0.02 ± 0.39	−0.02 ± 0.44	0.134
Coma 90 (μm)	−0.82 ± 0.40	−0.24 ± 0.35	<0.001	−0.74 ± 0.49	−0.64 ± 0.50	0.005
Trefoil 0 (μm)	0.05 ± 0.17	−0.01 ± 0.12	0.102	−0.01 ± 0.14	0.04 ± 0.12	0.102
Trefoil 30 (μm)	−0.04 ± 0.14	0.04 ± 0.16	0.049	0.08 ± 0.24	0.10 ± 0.20	0.851
Spherical aberration	−0.17 ± 0.19	−0.06 ± 0.16	0.001	−0.15 ± 0.15	−0.13 ± 0.14	0.400
6.0 mm zone						
RMS total (μm)	9.82 ± 3.85	5.95 ± 3.31	<0.001	9.26 ± 4.04	8.54 ± 4.19	0.001
RMS LOAs (μm)	9.56 ± 3.75	5.77 ± 3.23	<0.001	9.00 ± 3.87	8.28 ± 4.03	0.001
RMS HOAs (μm)	2.21 ± 0.89	1.41 ± 0.79	<0.001	2.15 ± 1.20	2.05 ± 1.22	0.209
Coma 0 (μm)	0.22 ± 0.89	0.07 ± 0.73	0.017	0.05 ± 1.00	−0.02 ± 1.14	0.357
Coma 90 (μm)	−1.65 ± 0.79	−0.79 ± 0.72	<0.001	−1.49 ± 1.16	−1.30 ± 1.21	0.093
Trefoil 0 (μm)	0.12 ± 0.36	0.02 ± 0.28	0.177	−0.01 ± 0.23	0.06 ± 0.26	0.199
Trefoil 30 (μm)	−0.14 ± 0.38	0.02 ± 0.33	0.039	0.10 ± 0.50	0.14 ± 0.42	0.735
Spherical aberration	−0.57 ± 0.59	−0.41 ± 0.51	0.020	−0.48 ± 0.49	−0.45 ± 0.47	0.586

TG-PRK, topography-guided photorefractive keratectomy; ACXL, accelerated corneal cross-linking; RMS, root mean square; LOAs, the lower-order aberrations; HOAs, the higher-order aberrations. Data were presented as mean ± standard deviation unless otherwise indicated. A *P* value of less than 0.05 was considered statistically significant.

Table 5 showed the changes of the densitometric values at the last follow-up visit. For both groups, the densitometric values within the central 0–2 and 2–6 mm significantly increased at the last follow-up visit (*p* < 0.001).

**Table 5:** Pre- and postoperative comparison of densitometric data for the TG-PRK plus ACXL group and the ACXL alone group at the last follow-up visit.

	TG-PRK plus ACXL group (*n* = 30)			ACXL alone group (*n* = 15)		
	Preoperative	Last follow-up	*p*-value	Preoperative	Last follow-up	*p*-value
0.0–2.0 mm	12.08 ± 1.03	16.20 ± 2.86	<0.001	12.41 ± 1.16	15.99 ± 2.13	<0.001
2.0–6.0 mm	10.80 ± 0.99	13.91 ± 2.22	<0.001	11.08 ± 1.00	14.33 ± 1.85	<0.001

TG-PRK, topography-guided photorefractive keratectomy; ACXL, accelerated corneal cross-linking. Data were presented as mean ± standard deviation unless otherwise indicated. A *P* value of less than 0.05 was considered statistically significant.

Table 6 compared the changes in the topography, aberrometric and the densitometric values between the two groups at the last follow-up visit. The analysis showed that compared with the ACXL alone group, the Kmax of the TG-PRK plus ACXL group showed a greater decline (*p* = 0.008). The declines of the total corneal aberrations, the corneal LOAs, the corneal HOAs and the vertical coma at the 4.0 mm and 6.0 mm zone of the TG-PRK plus ACXL group were significantly higher than those in the ACXL alone group (*p* < 0.001). The SA at the 2.0 mm and 4.0 mm zone of the TG-PRK plus ACXL group showed a greater decrease, but the changes of SA at the 6.0 mm zone showed no significant difference between the two groups (*p* > 0.05). The changes in the densitometric values at the last follow-up visit showed no significant differences between two groups (*p* > 0.05).

**Table 6:** Comparison of the changes in topography, aberrometric and densitometric data between the TG-PRK plus ACXL group and the ACXL alone group at the last follow-up visit.

	TG-PRK plus ACXL group (*n* = 30)	ACXL alone group (*n* = 15)	*p*-value
Topography			
ΔK1 (D)	0.53 ± 0.78	0.07 ± 0.79	0.067
ΔK2 (D)	0.65 ± 1.29	0.26 ± 1.10	0.325
ΔKmax (D)	3.06 ± 2.68	0.95 ± 1.58	0.008
2.0 mm aberrometric values			
ΔRMS total (μm)	0.24 ± 0.26	0.08 ± 0.20	0.051
ΔRMS LOAs (μm)	0.22 ± 0.26	0.08 ± 0.20	0.070
ΔRMS HOAs (μm)	0.09 ± 0.06	0.01 ± 0.02	<0.001
ΔComa 0 (μm)	0.01 ± 0.05	0.01 ± 0.02	0.841
ΔComa 90 (μm)	−0.11 ± 0.06	−0.02 ± 0.02	<0.001
ΔTrefoil 0 (μm)	0.01 ± 0.04	−0.01 ± 0.02	0.020
ΔTrefoil 30 (μm)	−0.01 ± 0.04	0.00 ± 0.04	0.358
ΔSpherical aberration	−0.01 ± 0.02	0.00 ± 0.01	0.017
4.0 mm aberrometric values			
ΔRMS total (μm)	1.54 ± 1.09	0.36 ± 0.43	<0.001
ΔRMS LOAs (μm)	1.47 ± 1.06	0.35 ± 0.45	<0.001
ΔRMS HOAs (μm)	0.50 ± 0.28	0.08 ± 0.12	<0.001
ΔComa 0 (μm)	0.08 ± 0.24	0.04 ± 0.10	0.539
ΔComa 90 (μm)	−0.58 ± 0.33	−0.10 ± 0.12	<0.001
ΔTrefoil 0 (μm)	0.06 ± 0.20	−0.06 ± 0.12	0.044
ΔTrefoil 30 (μm)	−0.08 ± 0.21	−0.01 ± 0.26	0.356
ΔSpherical aberration	−0.11 ± 0.16	−0.02 ± 0.08	0.012
6.0 mm aberrometric values			
ΔRMS total (μm)	3.87 ± 2.46	0.72 ± 0.66	<0.001
ΔRMS LOAs (μm)	3.80 ± 2.43	0.72 ± 0.65	<0.001
ΔRMS HOAs (μm)	0.80 ± 0.50	0.10 ± 0.30	<0.001
ΔComa 0 (μm)	0.15 ± 0.33	0.06 ± 0.25	0.357
ΔComa 90 (μm)	−0.86 ± 0.56	−0.19 ± 0.41	<0.001
ΔTrefoil 0 (μm)	0.10 ± 0.41	−0.08 ± 0.22	0.117
ΔTrefoil 30 (μm)	−0.17 ± 0.42	−0.04 ± 0.42	0.337
ΔSpherical aberration	−0.16 ± 0.36	−0.03 ± 0.22	0.204
Densitometric values			
Δ0.0–2.0 mm	−4.12 ± 2.87	−3.58 ± 2.32	0.531
Δ2.0–6.0 mm	−3.11 ± 2.13	−3.25 ± 2.11	0.832

TG-PRK, topography-guided photorefractive keratectomy; ACXL, accelerated corneal cross-linking; K1, flat keratometry; K2, steep keratometry; Kmax, the maximal keratometry of the anterior corneal surface; RMS, root mean square; LOAs, the lower-order aberrations; HOAs, the higher-order aberrations. Data were presented as mean ± standard deviation unless otherwise indicated. A P value of less than 0.05 was considered statistically significant.

In the TG-PRK plus ACXL group, 14 eyes (46.7%) were categorized into the severe subgroup, and 16 eyes (53.3%) were categorized into the mild to moderate subgroup. In the ACXL alone group, 7 eyes (46.7%) were categorized into the severe subgroup, and 8 eyes (53.3%) were categorized into the mild to moderate subgroup. Subgroup analysis showed the significant difference between the mean change of the Kmax of the TG-PRK plus ACXL group and the ACXL alone group in the severe subgroup (*p* = 0.024), while no significant difference was found in the mean change of the topographic parameters in the mild to moderate subgroup (all *p* > 0.05). In both subgroups, the changes of the total corneal aberrations, the corneal LOAs, the corneal HOAs and the vertical coma at the 4.0 mm and the 6.0 mm zone differed significantly between the two procedures (all *p* < 0.05). No significant difference was found in the changes of the densitometric values in either subgroup (all *p* > 0.05; Supplementary Table S1).

Central corneal haze had been noted in 20 eyes (66.7%) of the TG-PRK plus ACXL group and 9 eyes (60.0%) of the ACXL alone group postoperatively. The haze had regressed completely in all patients at the last follow-up visit, but the severity and duration of the haze differed between the two groups. In the ACXL alone group, the haze ranged from 0.5 to 1 in all patients, and regressed within 1 year postoperatively. However, in the TG-PRK plus ACXL group, 3 eyes had exhibited a haze score of 2. The haze score ranged from 0.5 to 1 in other eyes. Besides, the haze could be observed in 6 eyes (20.0%) of the TG-PRK plus ACXL group at the 1-year postoperative follow-up visit, of which the haze regressed completely by 2 years post-operatively. No other severe complications were observed.

## 4 Discussion

This study prospectively compared the long-term outcome, especially the corneal aberrometric results of combined TG-PRK with ACXL and ACXL alone. The results of this study suggested that the combined procedure had the better long-term outcome and could be beneficial for the improvement in the corneal HOAs.

CXL was first reported by Wollensak et al. (6), and its treatment plan was named as the Dresden protocol. It is considered as the standard treatment for progressive keratoconus due to the long-term efficacy and safety (26, 27). More recently, different protocols have been proposed. The accelerated protocol shortened the operation time by increasing the irradiation intensity (5). It had been proved to have similar efficacy and safety compared with the standard protocol (28). In our study, we applied the ACXL at 30 mW/cm^2^ for 4 min. It maintained the adequate total dosage while improving the operating efficiency, therefore minimized the risk for complications.

For keratoconus patients, the progressive protrusion of the cornea caused the increased corneal irregularity, which led to the high myopia and irregular astigmatism (29). The stiffening effect generated by the CXL would confer a certain degree of the corneal curvature regularization. However, in fact, most patients who underwent CXL still complained about the blurred vision due to the significant residual corneal irregularity (19). Accordingly, the concept of “CXL plus” was proposed, referring to the combining refractive procedures that aims to further regularize the cornea (30). Kanellopoulos et al. first described a sequential treatment combining TG-PRK and CXL (9). Thereafter, Krueger and Kanellopoulos proposed a simultaneous protocol combining TG-PRK and the high-influence CXL, which was named “Athens protocol.” It is still the classical approach for corneal regularization in keratoconus (10, 11). As an effective treatment option, the Athens protocol showed the long-term safety, stability as well as the long-lasting improvement of the visual function (31). The conventional Athens protocol planned to correct 70% of the cylinder and less than 70% of the sphere, so as not to exceed the maximal stromal removal of 50 μm. Besides, the optical zone was limited to 5.5 mm (11, 32). However, in our study, only partial cylinder was actively corrected. The optical zone was appropriately expanded under the limit of the maximal stromal removal. We expected to get a greater improvement of the visual quality while preserving as much corneal tissue as possible. In previous comparative studies, the UDVA of the patients who underwent the Athens protocol procedure markedly improved in the long-term follow-up (31). Besides, compared with the CXL alone, combined TG-PRK with CXL showed more significant improvement in the UDVA (15–18). However, our study found no significant improvement in UDVA in either group. This was likely due to the fact that the corneal ablation was performed in our study with no sphere correction and partial cylinder correction, therefore the pre-existing refractive error remained mostly unchanged after surgery in both groups. It is worth pointing out that although the combined procedure was performed with a certain cylinder correction, our study found no significant difference in the refractive values before and after the surgery. On the one hand, the limited sample size of our study might mask the minor discrepancy in the preoperative and postoperative cylinder values. On the other hand, the corneal morphology of the keratoconus patients inevitably altered during the long-term follow-up, which made it difficult to ensure the stability of the laser ablation. The vector analysis also showed the inaccuracy of the refractive correction after the long-term follow-up.

The results of our study demonstrated the long-term efficacy of TG-PRK procedure in improving the CDVA. CDVA improved significantly in the TG-PRK plus ACXL group, whereas it remained unchanged in the majority of the patients of the ACXL alone group. This matched the results of preceding studies (15–18). Compared with the ACXL alone, the combined procedure further regularized the anterior corneal surface, therefore eliminated most of the irregular astigmatism unable to be fully corrected by the spectacles.

The laser ablation caused a significant decrease in the pachymetry of the TG-PRK plus ACXL group. Despite this, in our study, the combined protocol showed the long-term stability. We found a significant decrease in K1, K2 and Kmax of the TG-PRK plus ACXL group after the long-term follow-up. The Kmax of the ACXL alone group also showed a significant decrease, but the reduction was significantly lower than the TG-PRK plus ACXL group. On the one hand, the TG-PRK procedure remodeled the anterior surface of cornea, and therefore led to the decrease in keratometric values (19). A previous study showed that the improvement of the keratometric values after the combined procedure was maintained in the long-term follow-up (31). On the other hand, the stiffening effect of the CXL had been reported to cause the flattening of the cone apex and the steepening of the part of the cornea symmetrically opposite the cone (33). Several studies had compared the reduction in keratometric values of the TG-PRK plus CXL with CXL alone. Among them, some studies showed that the combining treatment was advantageous in the improvement of the keratometric values (15, 16), while the other showed no significant difference (17). However, a latest meta-analysis clearly showed that compared with CXL alone, TG-PRK plus CXL showed a significant reduction in keratometric values such as Kmax (18). Our findings supported this result, and further showed that the differences in the changes of the Kmax mainly occurred in the severe subgroup. This might suggest that compared with mild to moderate keratoconus patients, those with the severe keratoconus might benefit more from the TG-PRK plus ACXL procedure. Moreover, the better results in the keratometric values of the combined treatment also corresponded to the significantly greater improvement in CDVA and aberrometric values.

Our study revealed the long-term changes in the aberrometric values of two treatments. In our study, the total corneal aberrations and the corneal LOAs significantly decreased at the 4.0 mm and 6.0 mm corneal zone of the both groups. The measurement of the corneal HOAs showed that the total corneal HOAs and the vertical coma at each corneal zone improved significantly in the TG-PRK plus ACXL group at the last follow-up visit. In the ACXL alone group, the total corneal HOAs and the vertical coma of the 2 mm and 4 mm zone improved significantly. Besides, the improvements in the total corneal aberrations, the corneal LOAs, the corneal HOAs and the vertical coma of the TG-PRK plus ACXL group were significantly higher than the ACXL alone group. It had been well-established that the total corneal aberrations, the corneal LOAs, the corneal HOAs and the coma-like aberrations significantly increased in the keratoconus eyes, and the change of the aberrations occurred predominantly in the anterior corneal surface (34, 35). Besides, the higher-order aberrations of the keratoconus eyes mainly consisted of the coma-like aberrations, which was caused by the misalignment of the corneal apex and the other optic elements (34). Previous studies had demonstrated that CXL might potentially improve the corneal HOAs and the total coma of the anterior cornea (36, 37). The TG-PRK procedure further regularized the morphology of the anterior corneal surface and decreased the corneal asymmetry, and therefore effectively reduced the aberrometric values, especially the coma-like aberrations (38). Iselin et al. reported that compared with the CXL alone, the combined procedure showed a significant decrease of the total corneal aberrations, the corneal LOAs, the corneal HOAs and the coma-like aberrations at 12 months of follow-up (15). Alessio et al. reported that the total corneal aberrations and the coma-like aberrations significantly decreased after 24 months of follow up in the TG-PRK plus CXL group (16). The results of our study matched these findings, and further illustrated that the improvement of the coma-like aberrations was mainly due to the improvement of the vertical coma. This might be attributed to the decrease of the vertical asymmetry of the cornea. Moreover, in both subgroups, the changes in the total corneal HOAs and the vertical coma of the TG-PRK plus ACXL group were significantly higher than the ACXL alone group. From the perspective of the improvement in the corneal HOAs, patients within either subgroup could benefit from the combined procedure. In our study, the SA showed a significant decrease in the TG-PRK plus ACXL group, and the significant difference with the ACXL alone group in the postoperative SA change was only detected in the 2 mm and 4 mm zone. Our previous study revealed a similar result (39). A few other studies supported our observations, showing a significant decrease in the SA after TG-PRK combined with ACXL (14, 40). The decrease of the SA over a certain range might correspond to the different software with ablation algorithms, while the improvement of the SA over a wider range might be caused by the flattening of the central cornea.

Our study demonstrated the long-term safety of the two treatments. Transient corneal haze is a common complication of the CXL, and it usually resolves within 1 year (41). In the combined TG-PRK with CXL surgery, the ablation of the Bowman layer leads to a greater effect of the CXL treatment on the anterior corneal stromal (17, 42). Besides, the PRK procedure involves the disruption of the basement membrane and underlying stroma, and the subsequent inflammatory response is also the crucial factor that leads to corneal opacity (43). The previous studies had reported a high incidence of the corneal haze after the combined TG-PRK with CXL surgery (44, 45). Few studies had compared the occurrence of the corneal haze between the two treatments. Alessio et al. reported that 7 of 17 eyes in the TG-PRK plus CXL group and 4 of 17 eyes in the CXL alone group had developed central corneal haze during 24 months of follow-up (16). Singal et al. showed that compared with CXL alone, the corneal haze of the eyes in the TG-PRK plus CXL group persisted for a longer duration (46). In concordance, in the TG-PRK plus ACXL group of our study, the corneal haze occurred with the higher severity and the slower regression. Even so, the incidence of the corneal haze was similar in both groups, and the corneal haze had regressed completely by the last follow-up visit. Therefore, in the long term, the safety of both treatments was similar.

The severity of the corneal haze can be quantitatively assessed by corneal densitometry analysis with the Scheimpflug technology (47). Studies evaluating the long-term densitometry changes after the CXL were scarce, and the existing studies had showed the inconsistent results (48, 49). Iselin et al. had reported an equivalent rise in densitometry values of the TG-PRK plus CXL and CXL alone at 12 and 24 months postoperatively (15). Our study provided a similar long-term outcome. At the last follow-up visit, both groups in our study showed an increase in the densitometry values of the central 2 mm zone and the adjacent 2.0 to 6.0 mm ring. No significant differences were found between the changes of the values of both groups, indicating the similar long-term safety. There were many factors that can influence the long-term outcome of the corneal densitometry, such as different surgical protocols, age of the patients and the severity of keratoconus (47). Hence, more quantitative evaluation is required to reveal the natural history of the corneal haze after the two treatments.

In this study, mitomycin C was not used during the TG-PRK procedure. It had been demonstrated that the adjunctive use of mitomycin C can effectively prevent the occurrence of the post-PRK corneal haze (43). However, the use of the mitomycin C in the combined CXL procedures remained controversial (50). Despite the fact that the mitomycin C was used in the conventional Athens protocol, it might increase the incidence of the corneal haze after the CXL (51). Kymionis et al. found that the CXL procedure itself could inhibit the regeneration of the keratocyte in the corneal anterior stromal (52). Therefore, additional use of the mitomycin C was not necessary (11).

There were also some limitations in our study. Firstly, the main outcome of our study was the aberrometric values provided by the Pentacam. The primary data Pentacam obtains is the corneal elevation, according to which corneal aberrations are calculated indirectly. The aberrometric values differed significantly between most aberrometers (53). Therefore, more comprehensive quantitative and instrumental studies are required to verify our results. Secondly, the corneal wavefront aberration parameters could not fully describe the visual quality of the patients. The aberrations of the entire eye and a subjective visual quality questionnaire should be included in future studies to enrich the analysis. Thirdly, owing to the limited sample size, the subgroup analysis might be underpowered to detect the significant differences between groups. Fourthly, due to the design of our study, patients involved in our study were relatively mild in the severity of keratoconus and had adequate corneal thickness. For patients with more severe keratoconus, the long-term outcomes of the CXL were not within the scope of our study. The optimal treatment for these patients needs further investigation. Fifthly, due to the limited sample size, both eyes of some patients were included in this study. The between-eye correlation may affect our results.

## Data Availability

The raw data supporting the conclusions of this article will be made available by the authors, without undue reservation.
