# Supplementary material for: Topography-guided photorefractive keratectomy combined with accelerated corneal collagen cross-linking versus cross-linking alone for progressive keratoconus: a long-term prospective cohort study
Source: Front Med (Lausanne). 2024 Aug 12;11:1420264. doi: 10.3389/fmed.2024.1420264 (PMC11345261; doi:10.3389/fmed.2024.1420264)
Supplement: Supplementary file 1 [file Data_Sheet_1.PDF]

Supplementary Material

Table S1 Subgroup analysis of the changes of the topography, aberrometric and densitometric data in the TG-PRK plus ACXL group and the ACXL alone group at the last follow-up visit

	**Kmax ≥ 55D**		**P Value**	**Kmax < 55D**		**P Value**
	**TG-PRK plus ACXL group (n=14)**	**ACXL alone group (n=7)**		**TG-PRK plus ACXL group (n=16)**	**ACXL alone group (n=8)**	
Topography						
ΔK1 (D)	0.64 ± 0.58	0.01 ± 1.05	0.093	0.44 ± 0.93	0.11 ± 0.56	0.367
ΔK2 (D)	0.81 ± 1.38	0.18 ± 1.45	0.350	0.51 ± 1.23	0.32 ± 0.77	0.708
ΔKmax (D)	3.79 ± 3.14	1.07 ± 1.89	0.024	2.42 ± 2.11	0.85 ± 1.38	0.071
2.0mm aberrometric values						
ΔRMS total (μm)	0.31 ± 0.30	0.08 ± 0.23	0.099	0.17 ± 0.20	0.08 ± 0.19	0.308

ΔRMS LOAs (μm)	0.30 ± 0.30	0.08 ± 0.24	0.119	0.16 ± 0.19	0.08 ± 0.19	0.361
ΔRMS HOAs (μm)	0.11 ± 0.06	0.02 ± 0.02	0.001	0.07 ± 0.05	0.01 ± 0.02	0.001
ΔComa 0 (μm)	0.01 ± 0.06	0.01 ± 0.02	0.786	0.01 ± 0.04	0.01 ± 0.01	0.898
ΔComa 90 (μm)	-0.12 ± 0.07	-0.02 ± 0.01	<0.001	-0.10 ± 0.05	-0.02 ± 0.03	0.001
ΔTrefoil 0 (μm)	0.02 ± 0.05	-0.02 ± 0.03	0.061	0.00 ± 0.02	0.00 ± 0.01	0.419
ΔTrefoil 30 (μm)	-0.02 ± 0.04	0.01 ± 0.05	0.146	0.00 ± 0.03	-0.01 ± 0.04	0.686
ΔSpherical aberration	-0.02 ± 0.02	0.00 ± 0.01	0.046	0.00 ± 0.01	0.00 ± 0.00	0.597
4.0mm aberrometric values						
ΔRMS total (μm)	1.93 ± 1.09	0.37 ± 0.43	0.002	1.21 ± 1.01	0.35 ± 0.45	0.009
ΔRMS LOAs (μm)	1.84 ± 1.06	0.36 ± 0.46	0.002	1.15 ± 0.97	0.35 ± 0.48	0.013

ΔRMS HOAs (μm)	0.59 ± 0.28	0.10 ± 0.12	<0.001	0.43 ± 0.28	0.05 ± 0.12	0.001
ΔComa 0 (μm)	0.10 ± 0.29	0.02 ± 0.14	0.519	0.06 ± 0.19	0.06 ± 0.07	0.915
ΔComa 90 (μm)	-0.63 ± 0.36	-0.08 ± 0.08	<0.001	-0.53 ± 0.30	-0.12 ± 0.15	0.002
ΔTrefoil 0 (μm)	0.11 ± 0.27	-0.09 ± 0.17	0.080	0.02 ± 0.11	-0.02 ± 0.06	0.244
ΔTrefoil 30 (μm)	-0.17 ± 0.24	0.04 ± 0.30	0.099	0.00 ± 0.16	-0.06 ± 0.22	0.487
ΔSpherical aberration	-0.19 ± 0.20	-0.02 ± 0.11	0.060	-0.05 ± 0.09	-0.01 ± 0.04	0.325
6.0mm aberrometric values						
ΔRMS total (μm)	4.76 ± 2.47	0.82 ± 0.56	<0.001	3.09 ± 2.23	0.64 ± 0.76	0.001
ΔRMS LOAs (μm)	4.68 ± 2.46	0.80 ± 0.54	<0.001	3.02 ± 2.19	0.65 ± 0.76	0.001
ΔRMS HOAs (μm)	0.92 ± 0.50	0.17 ± 0.33	0.001	0.70 ± 0.50	0.04 ± 0.28	0.002

ΔComa 0 (μm)	0.21 ± 0.37	-0.05 ± 0.28	0.130	0.10 ± 0.28	0.16 ± 0.18	0.625
ΔComa 90 (μm)	-0.91 ± 0.62	-0.08 ± 0.36	0.004	-0.82 ± 0.51	-0.28 ± 0.45	0.021
ΔTrefoil 0 (μm)	0.22 ± 0.55	-0.10 ± 0.32	0.177	0.00 ± 0.18	-0.06 ± 0.10	0.383
ΔTrefoil 30 (μm)	-0.35 ± 0.50	0.03 ± 0.51	0.118	0.00 ± 0.26	-0.10 ± 0.34	0.480
ΔSpherical aberration	-0.33 ± 0.43	-0.05 ± 0.30	0.141	-0.02 ± 0.21	-0.01 ± 0.13	0.970
Densitometric values						
Δ0.0-2.0mm	-3.42 ± 2.17	-3.18 ± 3.41	0.848	-4.73 ± 3.32	-3.92 ± 0.70	0.365
Δ2.0-6.0mm	-2.80 ± 1.87	-2.81 ± 3.04	0.989	-3.38 ± 2.36	-3.64 ± 0.75	0.696

Kmax = the maximal keratometry of the anterior corneal surface; TG-PRK = topography-guided photorefractive keratectomy; ACXL = accelerated corneal cross-linking; K1 = flat keratometry; K2 = steep keratometry; Kmax = the maximal keratometry of the anterior corneal surface; RMS = root mean square; LOAs = the lower-order aberrations; HOAs = the higher-order aberrations.

Data were presented as mean ± standard deviation unless otherwise indicated. A P value of less than 0.05 was considered statistically significant.

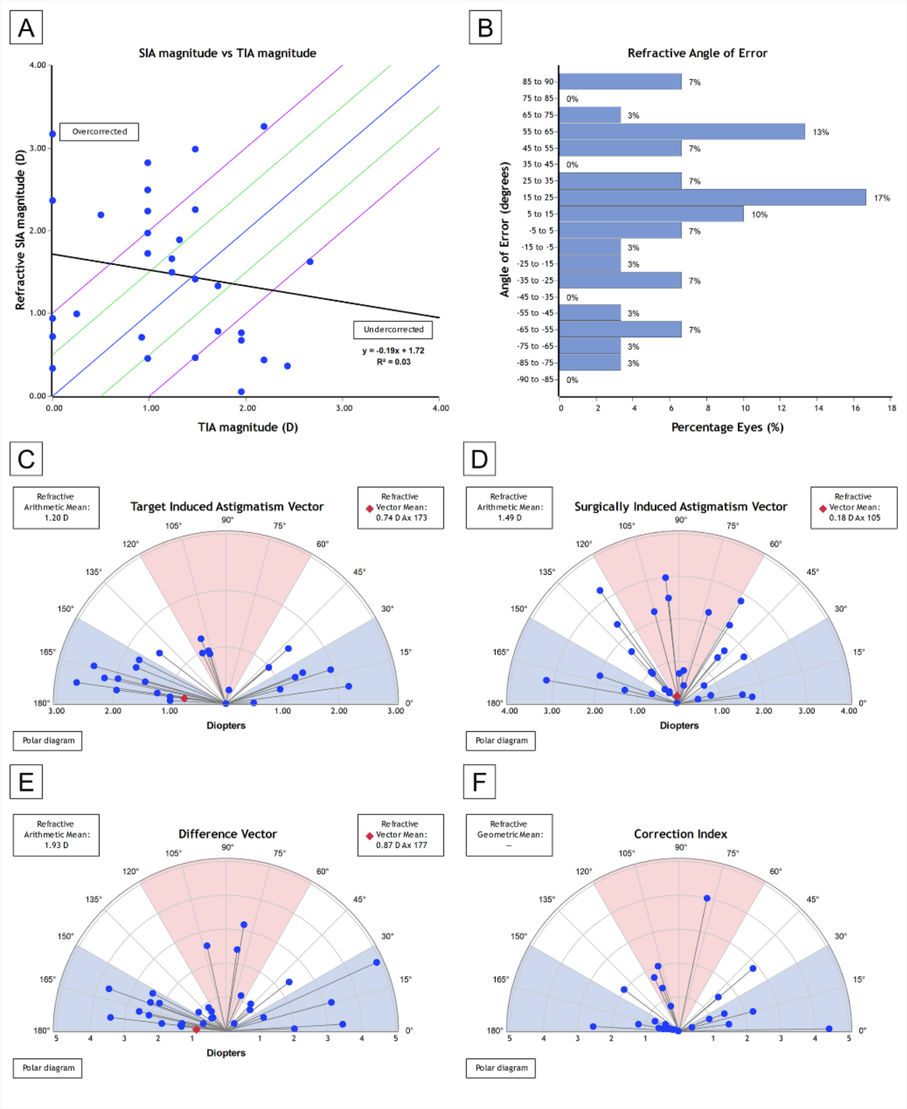
A
SIA magnitude vs TIA magnitude
Overcorrected
Undercorrected
y = -0.19x + 1.72
R² = 0.03
Refractive SIA magnitude (D)
TIA magnitude (D)
B
Refractive Angle of Error
Angle of Error (degrees)
Percentage Eyes (%)
C
Refractive Arithmetic Mean: 1.20 D
Target Induced Astigmatism Vector
Refractive Vector Mean: 0.74 D Ax 173
Diopters
Polar diagram
D
Refractive Arithmetic Mean: 1.49 D
Surgically Induced Astigmatism Vector
Refractive Vector Mean: 0.18 D Ax 105
Diopters
Polar diagram
E
Refractive Arithmetic Mean: 1.93 D
Difference Vector
Refractive Vector Mean: 0.87 D Ax 177
Diopters
Polar diagram
F
Refractive Geometric Mean: --
Correction Index
Polar diagram

Fig. S1 Postoperative vector analysis for the combined TG-PRK with ACXL procedure at the last follow-up visit. (A) Linear regression analysis of target induced astigmatism (TIA) versus surgically induced astigmatism (SIA). (B)Postoperative distribution of the angle of error. (C-F) Single-angle polar plots for the TIA, SIA, difference vector (DV) and correction index (CI).
